# Increased Incidence of New-Onset Diabetic Retinopathy in Individuals with COVID-19 in an Underserved Urban Population in the Bronx

**DOI:** 10.3390/diagnostics15151846

**Published:** 2025-07-22

**Authors:** Jai Mehrotra-Varma, Sonya Henry, Diane Chernoff, Andre Galenchik-Chan, Katie S. Duong, Shiv Mehrotra-Varma, Stephen H. Wang, Tim Q. Duong

**Affiliations:** 1 Department of Radiology, Montefiore Health System and Albert Einstein College of Medicine, Bronx, NY 10461, USA; jai.mehrotra-varma@icahn.mssm.edu (J.M.-V.); sonya.henry@einsteinmed.edu (S.H.); diane.chernoff@stonybrookmedicine.edu (D.C.); andre.galenchik-chan@stonybrookmedicine.edu (A.G.-C.); shiv.m.varma@gmail.com (S.M.-V.); stephen.wang@einsteinmed.edu (S.H.W.); 2 Department of Surgery, Beth Israel Deaconess Medical Center, Harvard Medical School, Boston, MA 02215, USA

**Keywords:** long COVID, post-acute sequelae SARS-CoV-2 infection (PASC), type 2 diabetes mellitus, diabetic complications, HbA1C, glucose

## Abstract

**Background/Objectives:** To investigate the incidence of new-onset diabetic retinopathy (DR) in individuals with pre-existing type 2 diabetes (T2D) up to 3 years post SARS-CoV-2 infection. **Methods:** This retrospective study consisted of 5151 COVID-19 and 5151 propensity-matched non-COVID-19 patients with T2D in the Montefiore Health System between 1 March 2020 and 17 January 2023. The primary outcome was new-onset DR at least 2 months after the index date up to 17 January 2023. Matching for index date between groups was also used to ensure the same follow-up duration. Hazard ratios (HRs) were computed, adjusted for competing risks. **Results:** T2D patients with COVID-19 had a higher cumulative incidence of DR than T2D patients. The unadjusted HR for COVID-19 status for developing new DR was 2.44 [1.60, 3.73], *p* < 0.001. The adjusted HR was 1.70 [1.08, 2.70], *p* < 0.05, and the adjusted HR for prior insulin use was 3.28 [2.10, 5.12], *p* < 0.001. Sex, ethnicity, and major comorbidities had no significant association with outcome. **Conclusions:** T2D patients who contracted COVID-19 exhibited a significantly higher risk of developing DR within three years post infection compared to propensity-matched controls. The increased incidence was primarily driven by greater pre-existing insulin usage and SARS-CoV-2 infection in the COVID-19 positive cohort.

## 1. Introduction

The acute clinical course of Coronavirus Disease 2019 (COVID-19) due to SARS-CoV-2 infection has been well-documented to be more severe in patients with pre-existing Type 2 diabetes mellitus (T2D), including higher rates of hospitalization, critical illness, and mortality [1,2,3,4,5,6,7,8,9]. Survivors of acute COVID-19 without any history of diabetes or pre-diabetes are also predisposed towards developing new-onset diabetes with an incidence higher than propensity-matched non-COVID controls [10,11,12,13,14]. It would not be surprising that some individuals with pre-existing T2D who were infected with SARS-CoV-2 may experience accelerated or more severe DM disease progression, including developing new diabetes-related complications, compared with individuals with pre-existing T2D who were not infected with SARS-CoV-2. 

There is evidence that the SARS-CoV-2 virus could directly infect insulin-producing β-cells in the pancreas and subsequently impair insulin secretion [15,16,17,18]. Systemic hypoxia, acute respiratory distress, pneumonia, sepsis, inflammatory responses, cytokine storm, and metabolic distress due to COVID-19 could also cause increased insulin resistance and metabolic decompensation acutely [19,20,21,22,23,24,25], which could lead to worse T2D disease progression and new diabetes-related complications. Although several studies have reported an increased incidence of persistent DM in COVID-19 patients compared to matched controls, it is unknown whether COVID-19 patients who have pre-existing T2D are more susceptible to developing new-onset diabetic retinopathy (DR) compared to non-COVID T2D patients. 

The goal of this study was to determine whether SARS-CoV-2 infection was associated with an increased incidence of new-onset DR among individuals with pre-existing T2D up to 3 years post infection. A comparison was made with propensity-matched pre-existing T2D individuals without COVID-19. Our data came from a large cohort of underserved inner-city population in the Bronx, an epicenter of the initial COVID-19 pandemic and subsequent surges of infections. A multivariate regression model was used to evaluate the relative risk.

## 2. Materials and Methods

### 2.1. Study Design

This retrospective cohort study was approved by the Einstein–Montefiore Institutional Review Board (2021-13658) with an exemption for informed consent and a HIPAA waiver and was performed in accordance with relevant guidelines and regulations. This includes following the protocol for reporting as per the Strengthening the Reporting of Observational Studies in Epidemiology (STROBE) guidelines. The Montefiore Health System includes multiple hospitals and outpatient clinics located primarily in the Bronx and surrounding areas. It is one of the largest healthcare systems in New York City, serving a large and diverse population which was heavily impacted by COVID-19 throughout the pandemic. 

### 2.2. Patient Cohorts

Electronic medical record data were extracted via OMOP data structures as described previously and other studies have been reported using previous versions of this dataset [26,27,28,29,30,31,32]. The data used were exported and queried as SQLite database files using the DB Browser for SQLite (https://sqlitebrowser.org, version 3.12.0). Data were obtained from 1 March 2020 to 9 January 2023. COVID-19 status was defined by a positive polymerase chain reaction (PCR) test. Contemporary controls were patients without a history of COVID-19 tests. The date of the positive COVID-19 test was used as index date. For non-COVID patients, the first visit to the Montefiore Health System after 1 March 2020 was used as the index date. Patients must have had a diagnosis of T2D (by ICD-10 code) prior to their index visit, but without DR diagnosis, and a return visit between 2 months to up to 3 years post index date. Non-COVID patients were propensity-matched (1:1) for age and sex, as well as for month of index visit. 

### 2.3. Clinical Variables

Demographic data (age, sex, ethnicity, race), pre-existing comorbidities (asthma, coronary artery disease (CAD), chronic obstructive pulmonary disease (COPD), chronic kidney disease (CKD), chronic heart failure (CHF), and hypertension), and body mass index (BMI), defined by ICD-10 diagnostic codes, were extracted. Average HbA1c was extracted over three time periods: up to 1 year pre index date, 14 days to 1 year post index date, and greater than 1 year post index date. For patients with multiple HbA1c tests over the designated time period, the average HbA1c for that patient was computed.

### 2.4. Outcomes

The primary outcome was new-onset DR by ICD-10 diagnosis code between 2 months and up to 3 years post index date. 

### 2.5. Statistical Analysis

Group comparison for categorical variables used χ^2^ exact tests, and for continuous variables, the independent *t*-test was used. *p*-values < 0.05 were considered statistically significant unless noted otherwise. Cumulative incidence functions of new-onset DR were plotted, and the Fine–Gray hazard model was used to estimate hazard ratio (HR) and 95% CI with adjustment for demographic and comorbidity variables. 

## 3. Results

### Cohort Description

**Figure 1:**
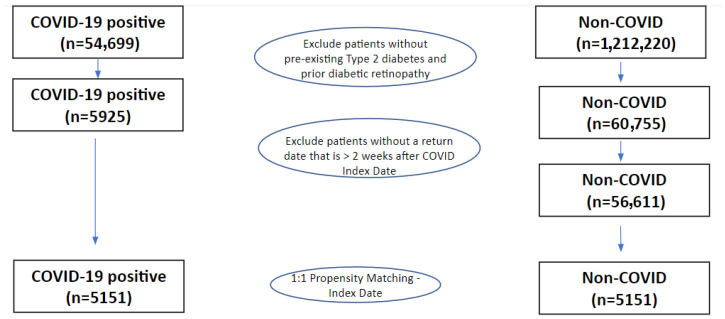
Patient selection flowchart.

Figure 1 shows the flowchart for patient selection. Between March 2020 and January 2023, 54,699 patients tested positive for COVID-19, of which 5925 had baseline T2D and no prior DR. During the same period, 1,212,220 patients were identified as non-COVID patients (controls), of which 60,755 had a history of pre-existing diabetes and no prior DR. The final cohort sizes after propensity-score matching for the index date and ensuring that patients returned to our health system at least 2 weeks after the index date were 5151 for COVID-19 and 5151 for non-COVID.

Table 1 summarizes the demographics and comorbidities for matched patients with and without COVID-19. There were slightly more female patients, Black patients, Hispanic patients, and fewer White patients in the COVID-19 positive cohort compared to controls after matching (all *p* < 0.05). The prevalence of baseline CHF, asthma, CAD, COPD, CKD, and hypertension were greater among COVID-19 patients compared to controls (all *p* < 0.05). BMI was not significantly different between groups (*p* < 0.05). HbA1c pre-index was not significantly different between groups (*p* < 0.05). 

There were 3117 (60.5%) patients hospitalized for COVID-19 and 636 (12.4%) had critical illness associated with acute COVID-19. 

HbA1c within a year and >1 year post infection was not significantly different between groups (*p* > 0.05). There were also no differences in HbA1c across different time points (*p* > 0.05). New-onset DR was higher among COVID-19 patients compared to controls (1.6% vs. 0.9%, *p* < 0.05, unadjusted). 

Table 2 shows the patients’ profiles of COVID-19 patients with and without DR. Patients with DR were younger, with an average age of 59.4 ± 12.4 compared to 64.6 ± 14.5 (*p* < 0.001). Fewer female patients were found to be DR+ than DR− (41.5% vs. 56.8%, *p* < 0.01). No statistically significant differences in race, ethnicity, or major comorbidities were found between groups (*p* > 0.05).

**Figure 2:**
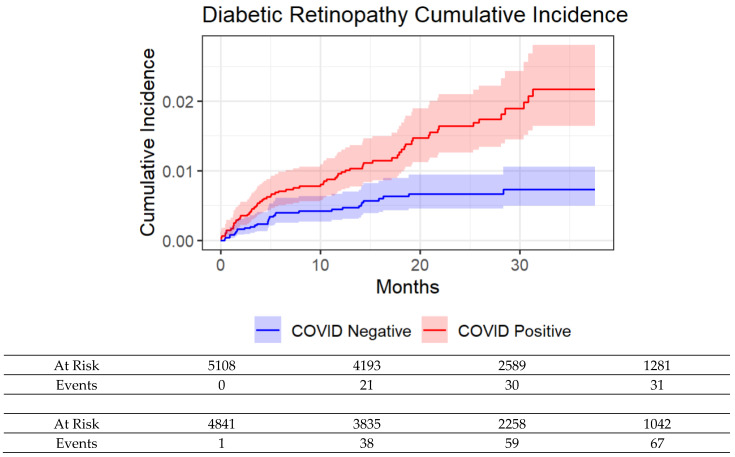
Cumulative incidence for developing diabetic retinopathy of COVID+ and COVID− patients with T2D. The cumulative incidence curve begins at 2 months post the index date (t = 0).

The cumulative incidence for developing DR was higher in the COVID-19 cohort compared to controls (Figure 2). Univariate and multivariate hazard ratios for developing new DR are shown in Table 3. The unadjusted HR for COVID-19 status for developing new DR was 2.44 [1.60, 3.73], *p* < 0.001. The adjusted HR was 1.70 [1.08, 2.70], *p* < 0.05, whereas the adjusted HR for prior insulin use was 3.28 [2.10, 5.12], *p* < 0.001. Female sex, race, ethnicity, or major comorbidities were generally not significantly associated with outcomes in either model (*p* > 0.05). Moreover, a subgroup analysis only involving patients with HbA1c values is shown in Table 4. The unadjusted HR for COVID-19 status for developing new DR was 2.01 [1.25, 3.22], *p* < 0.01. The adjusted HR was 1.55 [0.95, 2.52], *p* = 0.081, whereas the adjusted HR for prior insulin use was 3.43 [2.07, 5.67], *p* < 0.001.

## 4. Discussion

This study investigated the incidence of DR among patients with pre-existing T2D up to 3 years post SARS-CoV-2 infection in a large underserved inner-city patient population in the Bronx, an epicenter of the initial COVID-19 pandemic and subsequent surges of infections. The major findings are that the cumulative incidence of DR was higher in the COVID-19 cohort compared to non-COVID propensity-matched controls. The unadjusted HR for COVID-19 status for developing new DR was 2.44 [1.60, 3.73], *p* < 0.001. The adjusted HR was 1.70 [1.08, 2.70], *p* < 0.05, and the adjusted HR for prior insulin use was 3.28 [2.10, 5.12], *p* < 0.001. Our data suggest that the increased new-onset DR was primarily driven by greater pre-existing insulin usage and SARS-COV-2 infection. To our knowledge, this is the first study that showed patients with T2D who survive COVID-19 have a significantly higher risk of developing DR up to three years post infection compared to propensity-matched controls. 

Several reports have found the SARS-CoV-2 to be associated with increased new incidence of diabetes [12] and related complications [10,11,21]. One study performed in the Montefiore Health System concluded that COVID-19 infection increased risk of diabetes based on disease severity, as it was shown that new persistent DM was diagnosed in 16.7% of hospitalized COVID-19 patients versus 12% of hospitalized influenza patients but only 7.3% of non-hospitalized COVID-19 patients post infection [30]. This may be due to the viral destruction of insulin-producing β cells or the infection of adipose cells, resulting in enhanced insulin resistance as can be observed with the large doses of insulin sometimes required to manage hospital patients with diabetes [30]. Xu et al. [12] found that in hospitalized patients with a history of prediabetes, those infected with COVID-19 had a higher incidence of new DM 5 months post infection (14.75% vs. 7.51%, *p* < 0.001) compared to those not infected. This pattern of new DM incidence was not seen in non-hospitalized patients with and without COVID-19 (4.15% and 4.1%, *p* > 0.05). 

It is thus not surprising that SARS-CoV-2 infection could worsen T2D disease progression, which could lead to higher incidence of DR. In the multivariate regression model, we found that only COVID-19 status was significantly associated with a higher risk of developing DR. Sex, race, ethnicity, and major comorbidities (CHF, CKD, CAD, hypertension, COPD, and asthma) as covariates were not significantly associated with new DR. The exact mechanisms underlying how SARS-CoV-2 may trigger new DR are unknown. It is possible that SARS-CoV-2 infection and the ensuing hyperactive host-immune responses could worsen metabolic dysregulation, thereby promoting heightened insulin resistance and hyperglycemia [23]. The consequences of severe COVID-19 including systemic hypoxia, acute respiratory distress, hypercoagulation, hyperinflammation, metabolic stress, and cytokine storm could worsen diabetic conditions [32]. Specifically, elevated levels of inflammatory cytokines such as IL-1, IL-6 and TNF-alpha are found in patients with DR, and these same cytokines are activated in the body’s immune response to SARS-CoV-2 infection [33]. In addition, the viral destruction of pancreatic β-islet cells and invasion of adipose cells may result in greater relative insulin deficiencies [19,20,21,22], leading to new-onset DM and DM-related complications. The elevated cytokines and endothelial dysfunction associated with COVID-19 may cause damage to microvasculature. This inflammation may exacerbate the pathogenesis seen in DR, in which chronic breakdown of the blood–retinal barrier can lead to significant visual changes in vulnerable populations with diabetes [34,35,36]. Autoimmune/inflammatory syndrome induced by adjuvants (ASIA) syndrome and vaccine-induced immune thrombotic thrombocytopenia (VITT) which involve endothelial inflammation and thrombotic microvascular dysfunction may also exacerbate the retinal microangiopathy seen in certain patients [37,38,39]. Furthermore, in the multivariate model, we found the increased incidence was primarily driven by greater pre-existing insulin usage in the COVID-19 positive cohort, suggesting T2D disease severity played an important role in developing new DR. 

Pandemic circumstances such as the effects of isolation, psychosocial stress, reduced physical activity, unhealthy diet, weight gain, and interrupted care during the early pandemic could also contribute to the development of DM-related new clinical disorders [40,41,42]. Together, these direct and indirect effects of COVID-19 disease increase new-onset DR and other DM-related complications. 

### Limitations and Future Perspectives

This study is novel because it evaluated COVID-19 status as a risk factor for new-onset DR in T2D patients up to 3 years post infection. We compared the relative risk of COVID-19 status with other significant risk factors for DR. 

Our analysis had several limitations. Data were only included from returning patients to our health system (a predominant health system in the Bronx with over a dozen hospitals and few dozen clinics), and it is possible that returning patients were more likely to have severe COVID-19. However, our analysis included returning patients who came for any medical reason, not just COVID-19. It is also possible that some patients had previously undiagnosed diabetes or DR, which could result in misclassification. However, this misclassification likely occurred similarly in both experimental and control groups and should not alter our overall conclusions. Similarly, the COVID− cohort could have been tested positive elsewhere but were not recorded in our health system. Because the COVID− group is very large in sample size, the fraction of patients with COVID-19 but without a record in our system is likely relatively small. Surveillance bias is a potential concern, as patients with COVID-19 may have had more frequent interactions with the healthcare system, including ophthalmologic care. However, the absence of a marked increase in DR diagnoses shortly after infection in the COVID-19 cohort suggests that surveillance bias is unlikely to fully account for the findings. Outcomes could be affected by diabetes duration and insulin usage duration among others; however, these data were not reliably documented in the E nor readily extractable automatically. That said, we expected COVID+ and COVID− groups would have comparable durations at the population level. While HbA1c provides a measure of long-term glycemic control, it does not capture glycemic variability, time in range, or hypoglycemia, which may also impact diabetic retinopathy risk. This limitation makes it difficult to fully assess the relationship between glycemic control and retinopathy progression in our subgroup analysis with HbA1c data. Moreover, outcomes only included new-onset DR but did not attempt to stage DR. 

We relied on the accuracy of our electronic medical records, and, with data this size, there were bound to be some errors. This Montefiore Health System cohort is racially and ethnically diverse, and our findings might not be generalizable to other less diverse cohorts. A retrospective analysis could not provide causal inference but could only suggest associations which are affected by confounders.

This study also focused on evaluating the effects of insulin on outcomes but did not investigate other T2D medications with different doses and combinations. Furthermore, our patient cohort had high proportions of Hispanic and Black patients with their own risk profiles compared to national cohorts such as N3C [43]. The reference group of non-Hispanic White patients is also not the national average of Caucasians with respect to risks. This could affect why the values for Black patients were borderline insignificant in our model. Our findings need to be reproduced in larger and independent cohorts, including those that are less diverse. Future studies will also need to include longer follow-ups. Other new-onset T2D complications such as diabetic neuropathy and nephropathy, as well as other ophthalmological disorders such as conjunctivitis associated with COVID-19 should be explored. As with any retrospective study, there could be other unintended patient selection biases and latent confounds. 

## 5. Conclusions

Patients with T2D who survived COVID-19 had a higher risk for developing new-onset diabetic retinopathy compared to patients with T2D who did not contract COVID-19. This increased incidence is primarily driven by COVID-19 infection and duration of diabetes (with insulin acting as a proxy). Identifying risk factors for diabetic retinopathy may help with more effective screening and earlier diagnosis for at-risk patients.

## Figures and Tables

**Table 1:** Profiles of COVID+ and COVID− patients with T2D matched by index date. * *p* < 0.05, ** *p* < 0.01, and *** *p* < 0.001 between COVID+ and COVID− patients.

COVID-19	T2D Patients		
	COVID+ (*n* = 5151)	COVID− (*n* = 5151)	*p*-Values
Age, ±SD (yo)	64.5 ± 14.7	64.3 ± 14.3	0.48
Female, *n* (%)	2931 (56.9%) **	2787 (54.1%)	0.0046
Race, *n* (%)			
White	639 (12.4%) ***	1129 (21.9%)	<0.001
Black	1861 (36.1%) ***	1483 (28.8%)	<0.001
Other	2651 (51.5%) *	2539 (49.3%)	0.029
Ethnicity, *n* (%)			
Hispanic	2184 (42.4%) ***	1570 (30.5%)	<0.001
Non-Hispanic	2703 (52.5%)	2711 (52.6%)	0.89
Unknown	264 (5.1%) ***	870 (16.9%)	<0.001
Pre-existing comorbidities, *n* (%)			
CHF	1141 (22.2%) ***	387 (7.5%)	<0.001
CKD	1523 (29.6%) ***	734 (14.3%)	<0.001
CAD	150 (2.9%) ***	87 (1.7%)	<0.001
Hypertension	3644 (70.7%) ***	3223 (62.6%)	<0.001
COPD	385 (7.5%) ***	168 (3.3%)	0.001
Asthma	763 (14.8%) ***	414 (8.0%)	<0.001
BMI	30.6 ± 8.6	30.4 ± 7.4	0.21
HbA1c (1 year prior)	7.7 ± 2.1 *	7.6 ± 1.9	0.011
Prior Insulin Use	3197 (62.1%) ***	1905 (37.0%)	<0.001
COVID-19 Hospitalization	3117 (60.5%)	-	-
COVID-19 Critical Illness	636 (12.4%)	-	-
HbA1c, *n* (%)	3080 (59.8%) ***	4544 (88.2%)	<0.001
HbA1c (30 days to 1 year)	7.5 ± 2.0	7.4 ± 1.8	0.078
HbA1c (>1 year)	7.6 ± 1.9 **	7.4 ± 1.8	0.0053
New Diabetic Retinopathy	81 (1.6%) **	45 (0.87%)	0.0017
Patients with Visits >1 year Prior to Index Date	4955 (96.2%) ***	4726 (91.7%)	<0.001
Average Follow-up Time	638.675 ***	709.711	<0.001

**Table 2:** Profiles of COVID-19 patients with and without DR. ** *p* < 0.01, and *** *p* < 0.001 between COVID+ and COVID− patients.

COVID-19	COVID + Patients		
	DR+ (*n* = 81)	DR− (*n* = 5070)	*p*-Values
Age, ±SD (yo)	59.4 ± 12.5 ***	64.5 ± 14.7	<0.001
Female, *n* (%)	34 (42.0%) **	2897 (57.1%)	0.0088
Race, *n* (%)			
White	5 (6.17%)	634 (12.5%)	0.12
Black	28 (34.6%)	1833 (36.2%)	0.86
Other	48 (59.3%)	2603 (51.3%)	0.19
Ethnicity, *n* (%)			
Hispanic	37 (45.7%)	2147 (42.3%)	0.63
Non-Hispanic	41 (50.6%)	2662 (52.5%)	0.82
Unknown	3 (3.7%)	261 (5.2%)	0.74
Comorbidities, *n* (%)			
CHF	23 (28.4%)	1118 (22.1%)	0.22
CKD	26 (32.1%)	1497 (29.5%)	0.70
CAD	2 (2.5%)	148 (2.9%)	1.0
Hypertension	54 (66.7%)	3590 (70.8%)	0.49
COPD	3 (3.7%)	382 (7.5%)	0.28
Asthma	9 (11.1%)	754 (14.9%)	0.43
BMI	30.8 ± 9.0	30.6 ± 8.6	0.84
Hospitalization	58 (71.6%)	3059 (60.3%)	0.052
Critical Illness	15 (18.5%)	621 (12.2%)	0.13

**Table 3:** Multivariate Fine–Gray hazard ratio for developing DR in T2D patients.

	Univariate Fine–Gray Hazard Ratio				Multivariate Fine–Gray Hazard Ratio			
Variable	Hazard Ratio	5% CI	95% CI	*p*-Value	Hazard Ratio	5% CI	95% CI	*p*-Value
Prior Insulin Use	4.09	2.67	6.28	<0.001	3.28	2.10	5.12	<0.001
COVID-19 Positive	2.44	1.60	3.73	<0.001	1.70	1.08	2.70	0.023
Age	0.98	0.97	0.99	<0.001	0.98	0.97	0.99	<0.001
Female	0.72	0.49	1.06	0.098	0.73	0.49	1.09	0.13
Black	1.15	0.76	1.73	0.51	1.69	0.81	3.52	0.16
Other Race	1.22	0.82	1.81	0.32	1.50	0.69	3.29	0.31
Ethnicity	1.41	0.95	2.09	0.085	1.23	0.70	2.16	0.46
CHF	1.55	0.95	2.53	0.078	0.95	0.56	1.61	0.85
CKD	1.81	1.19	2.74	0.0054	1.40	0.86	2.30	0.18
CAD	1.37	0.44	4.30	0.59	1.13	0.36	3.58	0.84
Hypertension	1.20	0.78	1.84	0.40	1.25	0.77	2.01	0.36

**Table 4:** Multivariate Fine–Gray hazard ratio only including patients with HBA1C counts for developing DR in T2D patients.

	Univariate Fine–Gray Hazard Ratio				Multivariate Fine–Gray Hazard Ratio			
Variable	Hazard Ratio	5% CI	95% CI	*p*-Value	Hazard Ratio	5% CI	95% CI	*p*-Value
Prior Insulin Use	4.17	2.57	6.76	<0.001	3.43	2.07	5.67	<0.001
COVID-19 Positive	2.01	1.25	3.22	0.0038	1.55	0.95	2.52	0.081
Age	0.98	0.97	1.00	0.0049	0.98	0.97	0.99	<0.001
Female	0.80	0.52	1.21	0.29	0.87	0.56	1.33	0.51
Black	1.03	0.67	1.60	0.89	1.19	0.55	2.57	0.65
Other Race	1.05	0.69	1.61	0.81	1.05	0.46	2.41	0.90
Ethnicity	1.24	0.81	1.89	0.32	1.22	0.66	2.26	0.53
CHF	1.50	0.91	2.47	0.11	1.03	0.61	1.75	0.91
CKD	1.46	0.94	2.28	0.095	1.20	0.73	1.97	0.47
CAD	1.41	0.45	4.44	0.55	1.20	0.38	3.81	0.76
Hypertension	1.33	0.81	2.17	0.75	1.50	0.86	2.61	0.15
Average HbA1c	1.03	1.02	1.05	<0.001	1.02	1.01	1.04	<0.001

## Data Availability

The data underlying this article will be shared on reasonable request to the corresponding author.

## References

[1] Drucker D.J. (2021). Diabetes, obesity, metabolism, and SARS-CoV-2 infection: The end of the beginning. Cell Metab..

[2] Holman N., Knighton P., Kar P., O’Keefe J., Curley M., Weaver A., Barron E., Bakhai C., Khunti K., Wareham N.J. (2020). Risk factors for COVID-19-related mortality in people with type 1 and type 2 diabetes in England: A population-based cohort study. Lancet Diabetes Endocrinol..

[3] Shrestha D.B., Budhathoki P., Raut S., Adhikari S., Ghimire P., Thapaliya S., Rabaan A.A., Karki B.J. (2021). New-onset diabetes in COVID-19 and clinical outcomes: A systematic review and meta-analysis. World J. Virol..

[4] Zhu J.S., Ge P., Jiang C., Zhang Y., Li X., Zhao Z., Zhang L., Duong T.Q. (2020). Deep-learning artificial intelligence analysis of clinical variables predicts mortality in COVID-19 patients. J. Am. Coll. Emerg. Physicians Open.

[5] Rubino F., Amiel S.A., Zimmet P., Alberti G., Bornstein S., Eckel R.H., Mingrone G., Boehm B., Cooper M.E., Chai Z. (2020). New-Onset Diabetes in Covid-19. N. Engl. J. Med..

[6] Zhao Z., Chen A., Hou W., Graham J.M., Li H., Richman P.S., Thode H.C., Singer A.J., Duong T.Q. (2020). Prediction model and risk scores of ICU admission and mortality in COVID-19. PLoS ONE.

[7] Li X., Ge P., Zhu J., Li H., Graham J., Singer A., Richman P.S., Duong T.Q. (2020). Deep learning prediction of likelihood of ICU admission and mortality in COVID-19 patients using clinical variables. PeerJ.

[8] Hartmann-Boyce J., Rees K., Perring J.C., Kerneis S.A., Morris E.M., Goyder C., Otunla A.A., James O.A., Syam N.R., Seidu S. (2021). Risks of and From SARS-CoV-2 Infection and COVID-19 in People with Diabetes: A Systematic Review of Reviews. Diabetes Care.

[9] Gregg E.W., Sophiea M.K., Weldegiorgis M. (2021). Diabetes and COVID-19: Population Impact 18 Months into the Pandemic. Diabetes Care.

[10] Metwally A.A., Mehta P., Johnson B.S., Nagarjuna A., Snyder M.P. (2021). COVID-19-Induced New-Onset Diabetes: Trends and Technologies. Diabetes.

[11] Chandrashekhar Joshi S., Pozzilli P. (2022). COVID-19 induced Diabetes: A novel presentation. Diabetes Res. Clin. Pract..

[12] Xu A.Y., Wang S.H., Duong T.Q. (2023). Patients with prediabetes are at greater risk of developing diabetes 5 months postacute SARS-CoV-2 infection: A retrospective cohort study. BMJ Open Diabetes Res. Care.

[13] Ssentongo P., Zhang Y., Witmer L., Chinchilli V.M., Ba D.M. (2022). Association of COVID-19 with diabetes: A systematic review and meta-analysis. Sci. Rep..

[14] Rathmann W., Kuss O., Kostev K. (2022). Incidence of newly diagnosed diabetes after Covid-19. Diabetologia.

[15] van der Heide V., Jangra S., Cohen P., Rathnasinghe R., Aslam S., Aydillo T., Geanon D., Handler D., Kelley G., Lee B. (2022). Limited extent and consequences of pancreatic SARS-CoV-2 infection. Cell Rep..

[16] Wu C.T., Lidsky P.V., Xiao Y., Lee I.T., Cheng R., Nakayama T., Jiang S., Demeter J., Bevacqua R.J., Chang C.A. (2021). SARS-CoV-2 infects human pancreatic beta cells and elicits beta cell impairment. Cell Metab..

[17] Steenblock C., Richter S., Berger I., Barovic M., Schmid J., Schubert U., Jarzebska N., von Massenhausen A., Linkermann A., Schurmann A. (2021). Viral infiltration of pancreatic islets in patients with COVID-19. Nat. Commun..

[18] Tang X., Uhl S., Zhang T., Xue D., Li B., Vandana J.J., Acklin J.A., Bonnycastle L.L., Narisu N., Erdos M.R. (2021). SARS-CoV-2 infection induces beta cell transdifferentiation. Cell Metab..

[19] Huang C., Wang Y., Li X., Ren L., Zhao J., Hu Y., Zhang L., Fan G., Xu J., Gu X. (2020). Clinical features of patients infected with 2019 novel coronavirus in Wuhan, China. Lancet.

[20] Wang D., Hu B., Hu C., Zhu F., Liu X., Zhang J., Wang B., Xiang H., Cheng Z., Xiong Y. (2020). Clinical Characteristics of 138 Hospitalized Patients with 2019 Novel Coronavirus-Infected Pneumonia in Wuhan, China. JAMA.

[21] Lu J.Y., Babatsikos I., Fisher M.C., Hou W., Duong T.Q. (2021). Longitudinal Clinical Profiles of Hospital vs. Community-Acquired Acute Kidney Injury in COVID-19. Front. Med..

[22] Guo T., Fan Y., Chen M., Wu X., Zhang L., He T., Wang H., Wan J., Wang X., Lu Z. (2020). Cardiovascular Implications of Fatal Outcomes of Patients with Coronavirus Disease 2019 (COVID-19). JAMA Cardiol..

[23] Reiterer M., Rajan M., Gomez-Banoy N., Lau J.D., Gomez-Escobar L.G., Ma L., Gilani A., Alvarez-Mulett S., Sholle E.T., Chandar V. (2021). Hyperglycemia in acute COVID-19 is characterized by insulin resistance and adipose tissue infectivity by SARS-CoV-2. Cell Metab..

[24] Khunti K., Del Prato S., Mathieu C., Kahn S.E., Gabbay R.A., Buse J.B. (2021). COVID-19, Hyperglycemia, and New-Onset Diabetes. Diabetes Care.

[25] Shi S., Qin M., Cai Y., Liu T., Shen B., Yang F., Cao S., Liu X., Xiang Y., Zhao Q. (2020). Characteristics and clinical significance of myocardial injury in patients with severe coronavirus disease 2019. Eur. Heart J..

[26] Mehrotra-Varma S., Lu J.Y., Boparai M.S., Henry S., Wang S.H., Duong T.Q. (2024). Patients with type 1 diabetes are at elevated risk of developing new hypertension, chronic kidney disease and diabetic ketoacidosis after COVID-19: Up to 40 months’ follow-up. Diabetes Obes. Metab..

[27] Iosifescu A.L., Hoogenboom W.S., Buczek A.J., Fleysher R., Duong T.Q. (2022). New-onset and persistent neurological and psychiatric sequelae of COVID-19 compared to influenza: A retrospective cohort study in a large New York City healthcare network. Int. J. Methods Psychiatr. Res..

[28] Lu J.Y., Boparai M.S., Shi C., Henninger E.M., Rangareddy M., Veeraraghavan S., Mirhaji P., Fisher M.C., Duong T.Q. (2023). Long-term outcomes of COVID-19 survivors with hospital AKI: Association with time to recovery from AKI. Nephrol. Dial. Transpl..

[29] Lu J.Y., Buczek A., Fleysher R., Musheyev B., Henninger E.M., Jabbery K., Rangareddy M., Kanawade D., Nelapat C., Soby S. (2023). Characteristics of COVID-19 patients with multiorgan injury across the pandemic in a large academic health system in the Bronx, New York. Heliyon.

[30] Lu J.Y., Wilson J., Hou W., Fleysher R., Herold B.C., Herold K.C., Duong T.Q. (2023). Incidence of new-onset in-hospital and persistent diabetes in COVID-19 patients: Comparison with influenza. EBioMedicine.

[31] Hoogenboom W.S., Fleysher R., Soby S., Mirhaji P., Mitchell W.B., Morrone K.A., Manwani D., Duong T.Q. (2021). Individuals with sickle cell disease and sickle cell trait demonstrate no increase in mortality or critical illness from COVID-19—A fifteen hospital observational study in the Bronx, New York. Haematologica.

[32] Hoogenboom W.S., Pham A., Anand H., Fleysher R., Buczek A., Soby S., Mirhaji P., Yee J., Duong T.Q. (2021). Clinical characteristics of the first and second COVID-19 waves in the Bronx, New York: A retrospective cohort study. Lancet Reg. Health Am..

[33] Sathish T., Tapp R.J., Cooper M.E., Zimmet P. (2021). Potential metabolic and inflammatory pathways between COVID-19 and new-onset diabetes. Diabetes Metab..

[34] Bolla A.M., Loretelli C., Montefusco L., Finzi G., Abdi R., Ben Nasr M., Lunati M.E., Pastore I., Bonventre J.V., Nebuloni M. (2022). Inflammation and vascular dysfunction: The negative synergistic combination of diabetes and COVID-19. Diabetes Metab. Res. Rev..

[35] Yue T., Shi Y., Luo S., Weng J., Wu Y., Zheng X. (2022). The role of inflammation in immune system of diabetic retinopathy: Molecular mechanisms, pathogenetic role and therapeutic implications. Front. Immunol..

[36] Boss J.D., Singh P.K., Pandya H.K., Tosi J., Kim C., Tewari A., Juzych M.S., Abrams G.W., Kumar A. (2017). Assessment of Neurotrophins and Inflammatory Mediators in Vitreous of Patients with Diabetic Retinopathy. Investig. Ophthalmol. Vis. Sci..

[37] Cohen Tervaert J.W., Martinez-Lavin M., Jara L.J., Halpert G., Watad A., Amital H., Shoenfeld Y. (2023). Autoimmune/inflammatory syndrome induced by adjuvants (ASIA) in 2023. Autoimmun. Rev..

[38] Caldarelli M., Rio P., Giambra V., Gasbarrini A., Gambassi G., Cianci R. (2024). ASIA Syndrome: State-of-the-Art and Future Perspectives. Vaccines.

[39] Azzarone B., Veneziani I., Moretta L., Maggi E. (2021). Pathogenic Mechanisms of Vaccine-Induced Immune Thrombotic Thrombocytopenia in People Receiving Anti-COVID-19 Adenoviral-Based Vaccines: A Proposal. Front. Immunol..

[40] Ruissen M.M., Regeer H., Landstra C.P., Schroijen M., Jazet I., Nijhoff M.F., Pijl H., Ballieux B., Dekkers O., Huisman S.D. (2021). Increased stress, weight gain and less exercise in relation to glycemic control in people with type 1 and type 2 diabetes during the COVID-19 pandemic. BMJ Open Diabetes Res. Care.

[41] Khunti K., Aroda V.R., Aschner P., Chan J.C.N., Del Prato S., Hambling C.E., Harris S., Lamptey R., McKee M., Tandon N. (2022). The impact of the COVID-19 pandemic on diabetes services: Planning for a global recovery. Lancet Diabetes Endocrinol..

[42] Liang Y.Y., He Y., Wang J., Liu Y., Ai S., Feng H., Zhu C., Li H., Zhou Y., Zhang J. (2024). Social Isolation, Loneliness, and Risk of Microvascular Complications Among Individuals with Type 2 Diabetes Mellitus. Am. J. Kidney Dis..

[43] Kahkoska A.R., Abrahamsen T.J., Alexander G.C., Bennett T.D., Chute C.G., Haendel M.A., Klein K.R., Mehta H., Miller J.D., Moffitt R.A. (2021). Association Between Glucagon-Like Peptide 1 Receptor Agonist and Sodium-Glucose Cotransporter 2 Inhibitor Use and COVID-19 Outcomes. Diabetes Care.

